# Mammalian Target of Rapamycin Complex I (mTORC1) Activity in Ras Homologue Enriched in Brain (Rheb)-Deficient Mouse Embryonic Fibroblasts

**DOI:** 10.1371/journal.pone.0081649

**Published:** 2013-11-26

**Authors:** Marlous J. Groenewoud, Susan M. I. Goorden, Jorien Kassies, Wendy Pellis-van Berkel, Richard F. Lamb, Ype Elgersma, Fried J. T. Zwartkruis

**Affiliations:** 1 Molecular Cancer Research, Centre for Biomedical Genetics and Cancer Genomics Centre, University Medical Center Utrecht, Utrecht, The Netherlands; 2 Department of Neuroscience, ENCORE expertise center for neuro-developmental disorders, Erasmus MC University Medical Center, Rotterdam, The Netherlands; 3 Department of Molecular and Clinical Cancer Medicine, University of Liverpool, Cancer Research UK Centre, Liverpool, United Kingdom; Children's Hospital Boston & Harvard Medical School, United States of America

## Abstract

The Ras-like GTPase Rheb has been identified as a crucial activator of mTORC1. Activation most likely requires a direct interaction between Rheb and mTOR, but the exact mechanism remains unclear. Using a panel of Rheb-deficient mouse embryonic fibroblasts (MEFs), we show that Rheb is indeed essential for the rapid increase of mTORC1 activity following stimulation with insulin or amino acids. However, mTORC1 activity is less severely reduced in Rheb-deficient MEFs in the continuous presence of serum or upon stimulation with serum. This remaining mTORC1 activity is blocked by depleting the cells for amino acids or imposing energy stress. In addition, MEK inhibitors and the RSK-inhibitor BI-D1870 interfere in mTORC1 activity, suggesting that RSK acts as a bypass for Rheb in activating mTORC1. Finally, we show that this rapamycin-sensitive, Rheb-independent mTORC1 activity is important for cell cycle progression. In conclusion, whereas rapid adaptation in mTORC1 activity requires Rheb, a second Rheb-independent activation mechanism exists that contributes to cell cycle progression.

## Introduction

The mTORC1 complex plays a vital role in adapting cellular metabolism of mammalian organisms to changing conditions like progress through development, food intake, prolonged starvation or acute stress (reviewed in 1). Information about the availability of nutrients and growth factors is integrated by various proteins present in mTORC1 and transmitted via the kinase activity of mTOR (reviewed in 2). In mammalian cells, two functionally distinct protein complexes, mTORC1 and mTORC2, exist that share mTOR and LST8 as subunits. Raptor and PRAS40 are unique for mTORC1. The best-studied mTORC1 substrates are p70 S6 kinase1 (S6K) and 4E-BP1. S6K is activated upon phosphorylation by mTORC1 and its activity is crucial for cell growth. 4E-BPs inhibit translation (reviewed in 3) and proliferation by binding to the eukaryotic initiation factor eIF4E. mTORC1-mediated phosphorylation leads to a release of 4E-BP from eIF4E, overcoming this inhibition [3,4]. 

Multiple inputs emanating from various signaling pathways underlie the complex upstream regulation of mTORC1. The Ras-like GTPase Rheb is, when GTP-bound, a strong activator of mTORC1 [5,6]. This GTPase is under the negative control of the heterodimeric tumor suppressor complex TSC1/TSC2. TSC2 harbors a GTPase activating protein (GAP) domain that normally drives Rheb into the inactive GDP-bound state. Insulin receptor signaling downregulates TSC2 GAP-activity via direct phosphorylation of TSC2 by the phospatidylinositol-3-phosphate dependent kinase PKB [7,8]. Other kinases like ERK and RSK have also been shown to phosphorylate and thereby negatively regulate TSC2 [1]. Conversely, when energy supplies are limited a rise in AMP levels will activate the kinase AMPK that increases TSC2 activity [9]. The importance of TSC1/TSC2 in the control of mTORC1 is revealed in tuberous sclerosis patients, where functional loss of either TSC1 or TSC2 results in non-metastatic tumors (reviewed in 10). Indeed, in cells lacking TSC2 the fraction of GTP-bound Rheb is very high [11], which leads to constitutive mTORC1 activity. 

Raptor in the mTORC1 complex functions as a scaffold protein that by binding to so-called TOS-motifs in substrates like S6K and 4E-BP1 enhances their phosphorylation by mTORC1 [12]. A regulatory function for Raptor has also been suggested [13]. For example, TSC2 deficient cells remain sensitive to energy stress via phosphorylation of Raptor by AMPK, which inhibits mTORC1 [14]. Phosphorylation of Raptor at multiple, different residues by RSK [15] or ERK [16] on the other hand has been shown to positively regulate mTORC1. For ERK, these sites include S863 that acts like a priming site required for further phosphorylation of Raptor. However, this site has also been reported as a direct mTOR-phosphorylation site, indicating that mTOR, once activated, may influence the activity of the complex in which it resides [17,18]. PRAS40 is another mTORC1 complex member that has been proposed to act as a negative regulator of mTORC1 via binding to Raptor [19,20]. Release of PRAS40 is thought to be a two-step process, in which PRAS40 is first phosphorylated by PKB at S246 and then by mTORC1 at S183. Release of PRAS40 makes the TOS-binding motif in Raptor available for other substrates [21].

Apart from post-translational control, mTORC1 is also regulated by its subcellular localization. Depriving cells of amino acids interferes in insulin-induced mTORC1 activity even though more upstream components of the insulin signaling pathway are activated normally [22]. Since elevated mTORC1 activity in TSC2 knockout cells can also be suppressed by amino acid starvation, a TSC2-independent amino acid-sensing mechanism was postulated [23]. This mechanism was shown to involve the dimeric Rag GTPases (RagA/B and RagC/D) [24,25]. RagA/B is GTP-loaded in amino acid replete cells and targets the mTORC1 complex to lysosomes, where Rheb is located.

Despite its high level of conservation during evolution [26] species-specific functions for the mTOR pathway have been uncovered (reviewed in 27). Furthermore, genetic studies using conditional knockout mice demonstrate tissue-specific roles for the mTOR pathway [28] [29] [30]. A functional mTOR pathway is crucial for normal development, since targeted disruption of either mTOR or Raptor in mice results in early embryonic lethality [31] [32] [33]. It was therefore surprising that the phenotype of mice lacking Rheb was much milder compared to that of mice mutant for Raptor or mTOR [34] [35]. Here we set out to address the question if mTORC1 would still be active in the absence of Rheb using a panel of MEFs. The results demonstrate that while Rheb is required for the strong insulin and amino acid-induced mTORC1 activity, residual mTORC1 activity in Rheb-deficient cells is present, which is still subject to negative regulation by energy stress and amino acid withdrawal. This Rheb-independent mTORC1 activity helps to drive cell proliferation in a rapamycin-sensitive fashion. 

## Results

**Figure 1:**
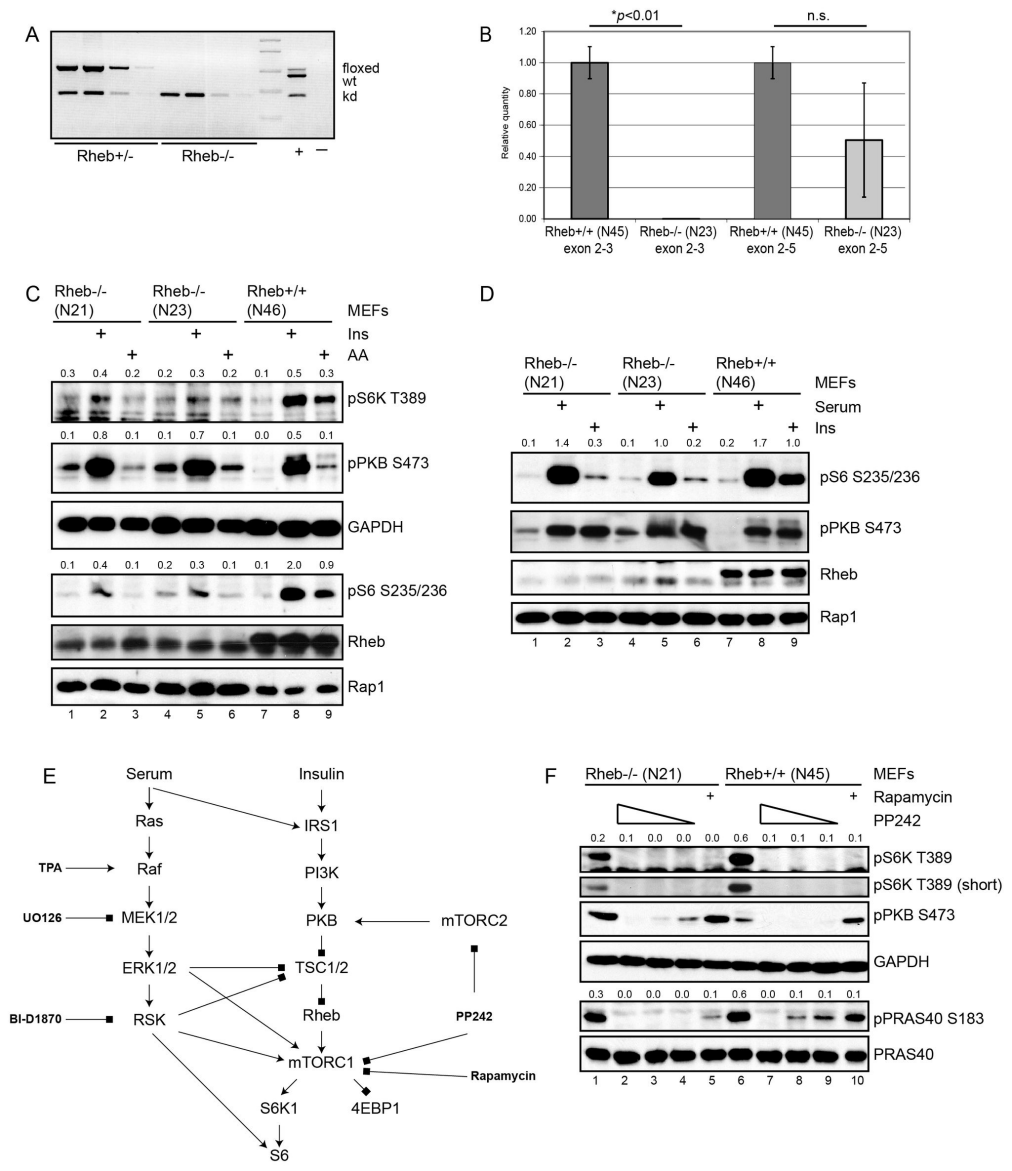
Characterization of Rheb-deficient cells (N21, N23) or control cells (N46, N45). **1a**. PCR on decreasing amounts of genomic DNA isolated from N46 cells (Rheb+/-), in which exon 3 from a single allele has been excised and N23 cells (Rheb-/-), in which exon 3 from both Rheb alleles have been removed. In the positive control lane (+) a mixture of DNA isolated from wild type animals, animals carrying the *loxP* sites surrounding exon 3 and Rheb-deficient embryos was used. In the negative control lane (-) no input DNA was used. **1b**. Q-PCR on mRNA isolated from Rheb-positive (N45; first and third bar) and Rheb-deficient (N23; second and fourth bar) cells using primers from exon 2 and 3 (first and second bar) or from exon 2 and 5 (third and fourth bar). **1c**. Analysis of mTORC1 activity in total lysates by Western blotting. Cells were serum starved overnight. and left untreated, stimulated with insulin for 30 minutes (Ins) or depleted for amino acids for two hours and then replenished with amino acids for 30 minutes (AA). Blots were probed with antibodies indicated on the right. The upper three panels represent reprobes of the same blot. GAPDH and Rap1 were used as loading controls. The immunoblots shown are representative for at least four experiments. Numbers on top of immunoblots indicate ratio Rap1 over pS6K T389. **1d**. Cells were serum starved overnight. and left untreated, stimulated with insulin for 30 minutes (Ins) or with serum. The immunoblots shown are representative of observations for at least two experiments. Rap1 was used as loading control. Numbers on top of immunoblots indicate ratio Rap1 over pS6K T389. **1e**. Overview of the PI3K and ERK pathway components converging on mTORC1 and indication of the inhibitors and stimuli used in this study. Arrows represent activation, squares indicate inhibition. **1f**. Asynchronously growing cells were either left untreated or treated with various concentrations of PP242 (2, 1 or 0.25 µM) or rapamycin (50 nM) for 60 minutes. Total cell lysates were probed with antibodies indicated on the right. The immunoblots shown are representative for at least two experiments. GAPDH was used as loading control. Numbers on top of immunoblots indicate the ratio of pS6K T389 relative to GAPDH.

We recently reported that genetic disruption of the Rheb results in embryonic lethality around day 12 [34]. Since this phenotype is much milder than that of mTOR or Raptor deletion, this result suggested that mTORC1 is active in the absence of Rheb or alternatively, that other signaling pathways can substitute for mTORC1. Therefore, we generated MEFs lacking Rheb. Spontaneously immortalized MEFs derived from a conditional Rheb knockout mouse in which exon 3 is surrounded by lox-sites [34] were infected with increasing amounts of Cre adenovirus. Four days after infection cells had strongly decreased levels of Rheb (Figure S1a). Subcloning of Ad-CRE-infected cells yielded both Rheb-deficient cell lines (e.g. N21, N23) and cells that had retained Rheb (e.g. N45, N46). Genomic analysis (Figure 1a), Q-PCR (Figure 1b, Figure S1b and S1c) and Western blotting (Figure 1c and d) demonstrated the complete absence of functional Rheb. Analysis of protein levels of various elements from the mTOR pathway did not reveal significant differences (Figure S1d). Furthermore, in cells growing in serum mTOR was localized on LAMP1-positive vesicles as detected by immunofluorescence (Figure S2a and S2b), while Rheb was found diffusely in the cytoplasm (Figure S2c). As expected, insulin stimulation of Rheb-deficient cells that had been serum starved only marginally activated mTORC1 as determined by probing cell lysates for phosphorylation of T389 of S6K, which is a direct target for mTORC1 (Figure 1c, compare lane 2 and 5 with 8). Similarly, mTORC1 in Rheb-deficient cells was virtually irresponsive to replenishment of amino acids following amino acid deprivation. Probing the same lysates for phosphorylation of S6, the downstream target of S6K, confirmed that loss of Rheb almost completely abolished insulin and amino acid-induced S6K activity. As expected, Rheb containing cells showed a strong enhancement of S6K activity in both situations. mTORC2 was normally activated by insulin in Rheb-deficient cells as judged from S473 phosphorylation of PKB. Under serum starved conditions the level of S473 was elevated as compared to Rheb-containing MEFs, most likely resulting from a lower level of negative feedback from S6K to IRS1 [36]. The localization of mTOR on LAMP1-positive vesicles was not severely affected by serum starvation or insulin stimulation, but appeared slightly more diffuse in amino-acid starved cells compared to cells stimulated with amino acids as has been reported [25] (Figure S2d and S2e). These results are in line with the documented role of Rheb in activating mTORC1 upon stimulation of cells with insulin or amino acids. 

Strikingly, we noticed that in Rheb-deficient cells grown in the presence of serum T389 phosphorylation of S6K was readily detectable, albeit at a lower level than in wild type cells (Figure 1f, compare lane 8 with 2 and 5). To test if this reflected mTORC1 activity, cells were treated with the ATP-competitive TOR inhibitor PP242 or with the mTORC1 inhibitor rapamycin (see Figure 1e for an overview of the mTORC1 pathway and inhibitors used in this study). As can be seen in Figure 1e, both inhibitors efficiently downregulated phospho-T389 levels (compare lane 1 with 2-5 and lane 6 to 7-10), while only PP242 affected PKB phosphorylation at S473 via inhibition of mTORC2. To see if other direct mTOR substrates were also phosphorylated in Rheb-deficient cells, we probed for phosphorylation of PRAS40 at S183. The levels of S183 PRAS40 phosphorylation correlated with those of S6K and were also clearly decreased by PP242 and rapamycin. PRAS40 levels were equal in Rheb-positive and -negative cells.

The marked degree of S6K phosphorylation in Rheb-deficient cell lines grown in serum prompted us to further investigate regulation of mTOR by serum. Therefore, we compared phosphorylation of S6 following insulin or serum stimulation of cells that had been starved of serum overnight. S6K activity was enhanced in both cell types following serum stimulation, although less pronounced in Rheb-deficient cells. In agreement with the data presented above, insulin stimulation only resulted in a significant increase in phosphorylation of S6 in wild type cells, while almost no effect was seen in Rheb-negative cells (Figure 1d). Thus, mTORC1 is still active in Rheb-deficient cells when grown in the continuous presence of serum or when stimulated with serum.

**Figure 2:**
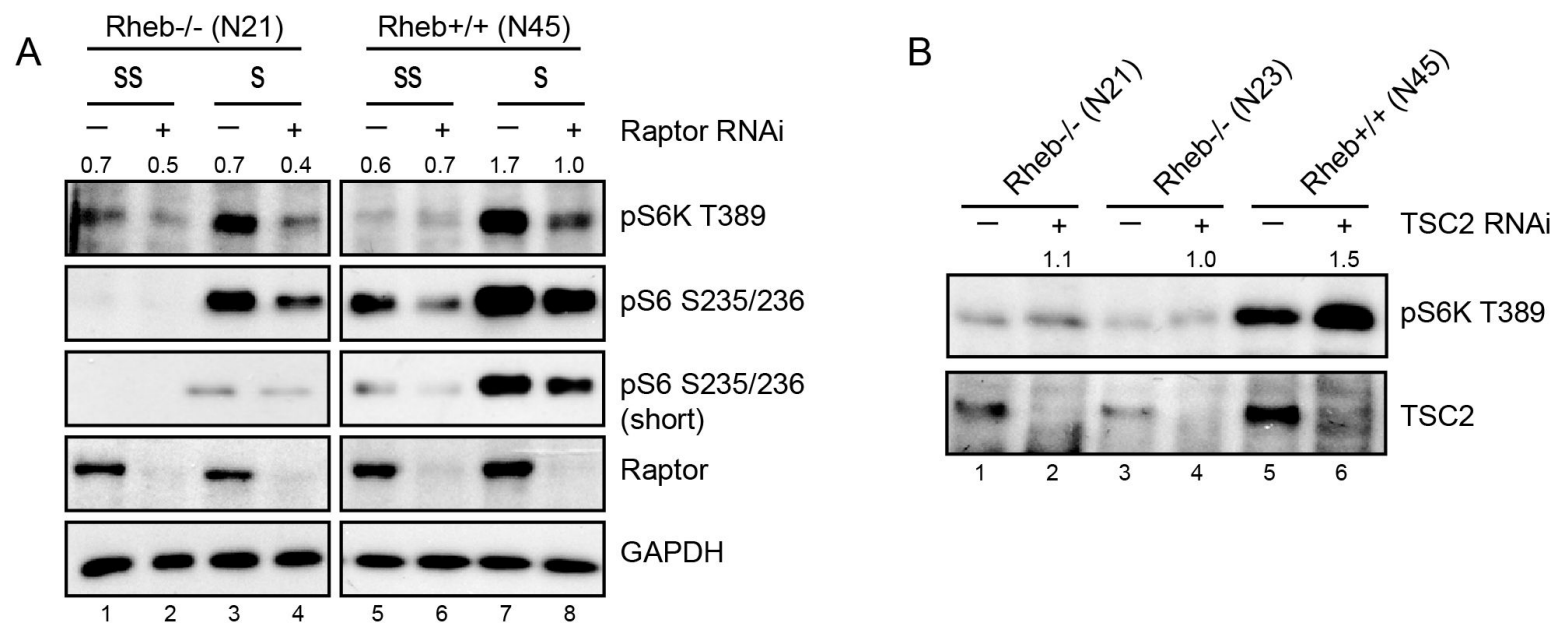
Western blot analysis of total lysates from Rheb-deficient cells (N21) and control cells (N45). **2a**. Cells treated with scrambled siRNA (-) or siRNA oligo’s directed against Raptor (+) were serum starved overnight (SS) or stimulated with serum for 90 minutes (S). Western blots were probed with the antibodies indicated on the right. A representative example of one out of two experiments is shown. Numbers on top of immunoblots indicate the ratio of pS6K T389 relative to GAPDH.. **2b**. Control (N45) and Rheb-deficient fibroblasts (N21, N23) were transfected with scrambled siRNA oligo’s or siRNA targeting TSC2. Total lysates of cells in the presence of serum were analyzed by Western blotting with the antibodies indicated on the right. The immunoblots shown are representative of observations for at least two experiments. Numbers on top of immunoblots indicate ratio pS6K T389 in cells with TSC2 RNAi relative to control.

To further substantiate that S6K phosphorylation in Rheb-deficient cells requires mTORC1 activity, we knocked down Raptor by RNAi. Indeed, a clear decrease in serum-induced S6K phosphorylation was seen in Rheb-deficient cells (Figure 2a, compare lane 3 with 4). Another hallmark of mTORC1 activity is inhibition by energy stress via activation of AMPK and subsequent phosphorylation of Raptor on S722 and S792 [14]. Therefore, cells were treated with phenformin or AICAR to activate AMPK. Both agents efficiently blocked T389 phosphorylation of S6K in asynchronously growing MEFs (Figure S3a, compare lane 1 with 2-3 and lane 5 with 6-7). Also osmotic stress induced by addition of sorbitol, completely abolished mTORC1 activity (compare lane 1 with 4 and 5 with 8). All three compounds clearly activated AMPK as judged from phosphorylation of S172 and induced phosphorylation of Raptor on S792. 

One obvious explanation for the residual mTORC1 activity in Rheb-deficient fibroblast would be the presence of RhebL1. This protein is highly similar to Rheb, but has a much more restricted expression pattern. Although there are no indications from the literature that suggest that RhebL1 would selectively respond to serum and not to insulin, we decided to knock down RhebL1. Knock down of RhebL1 using siRNA did not affect S6K activity (Figure S3b, lane 1 versus 2-3). Since efforts to detect endogenous RhebL1 protein in MEFs using commercial antibodies were unsuccessful, Q-PCR was used to judge the efficacy of RNAi (Figure S3c). As an alternative, we knocked down TSC2 that has been reported to act on RhebL1 [37] to see if this would lead to an increased S6K phosphorylation. While T389 phosphorylation of S6K is enhanced by TSC2 knock-down in control cells (Figure 2b, lane 5 versus 6), no such effect is seen in Rheb-deficient cell lines (lane 1 versus 2 and lane 3 versus 4). Together, these data further underscore that S6K phosphorylation in Rheb-deficient cells is mTORC1 dependent and sensitive to known negatively regulatory inputs other than TSC2. To see if a similar Rheb-independent level of mTORC1 activity could be detected in other asynchronously growing cells, we performed Rheb and RhebL1 knockdown experiments in A549 cells (Figure S3d). Despite an efficient knockdown of Rheb, which led to a reduction in phosphorylation of S6K T389 following insulin stimulation (lane 2 versus 4), a much less pronounced effect was seen in asynchronously growing cells (lane 7 versus 8). Additional knockdown of RhebL1 did not further decrease S6K phosphorylation (lane 6 and 9). These results support the idea that mTORC1 activity is partially Rheb-independent. 

**Figure 3:**
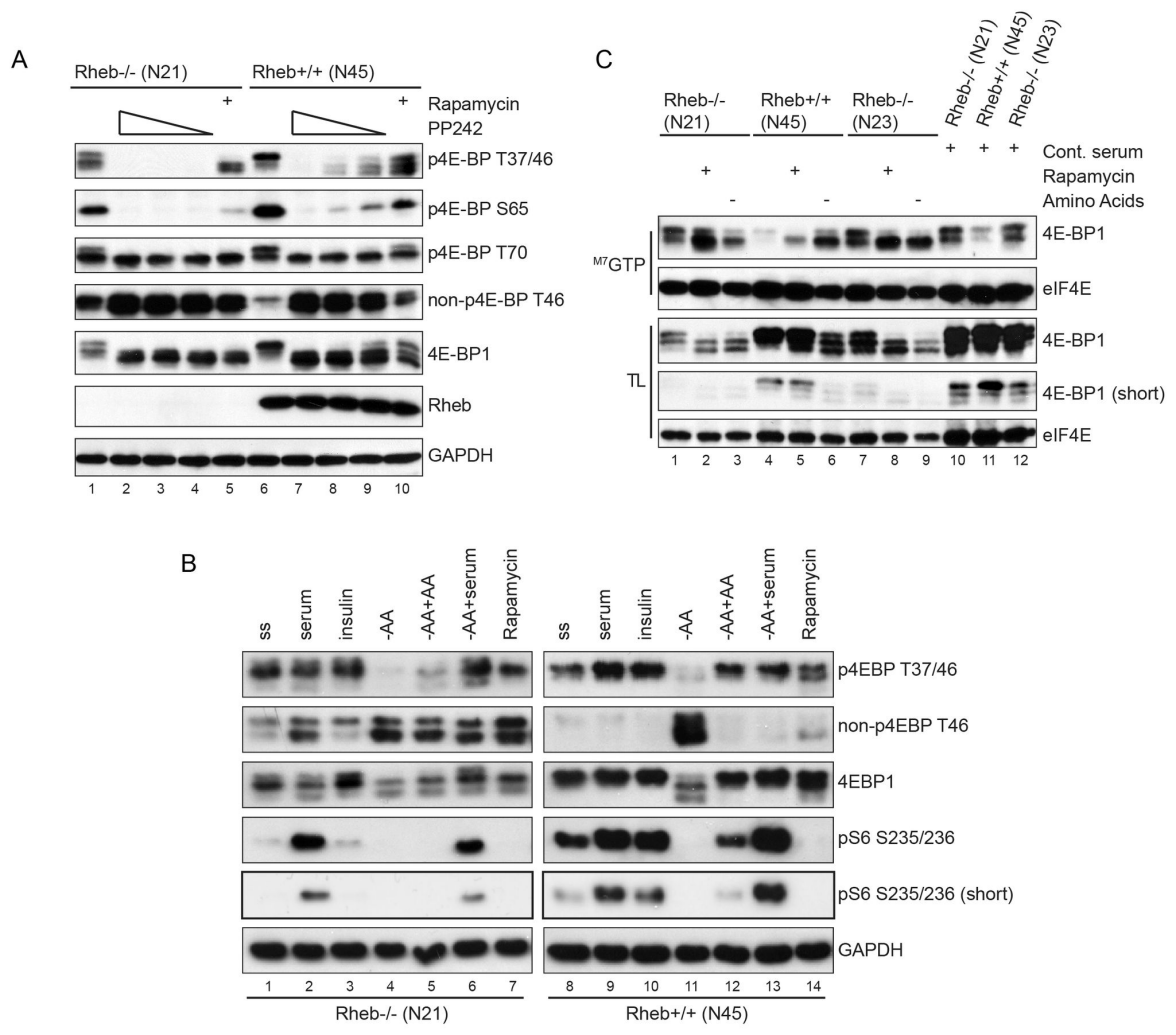
Effect of mTOR inhibition on 4E-BP1. **3a**. Asynchronously growing cells Rheb-deficient cells (N21) or control cells (N45) were either left untreated or treated with various concentrations of PP242 (2, 1 or 0.25 µM) or rapamycin (50 nM) for 60 minutes. Total cell lysates were probed with antibodies indicated on the right. **3B**. Rheb-deficient cells (N21) or control cells (N45) were serum starved overnight and stimulated with insulin, serum or rapamycin as indicated on the top. Alternatively cells were depleted for amino acids for two hours (-AA) after which the culture medium was reconstituted with amino acids (-AA +AA) for 30 minutes or serum (-AA +serum) for 90 minutes. Total lysates were analyzed by Western blot with the antibodies indicated on the right. **3C**. Association of 4E-BP1 with eIF4E from Rheb-deficient (N21, N23) or Rheb-positive (N45) cells. Cells serum starved overnight and then either treated with rapamycin for 1 hour or depleted for amino acids for two hours. Alternatively, they were grown in complete medium (lanes 10-12). Subsequently, cells were lysed and eIF4E plus associating proteins were pulled down using an ^m7^GTP-Sepharose pull-down assay. Isolated proteins (upper two panels) and total lysates (lower three panels) were analyzed by Western blotting with the antibodies indicated on the right. In all cases the immunoblots shown are representative of observations for at least two experiments.

4E-BP1 is another established mTOR target, which inhibits translation via its well-documented binding to eIF4E. 4E-BP1 is phosphorylated by mTOR at T37 and T46. However, in order to release 4E-BP1 from eIF4E-binding phosphorylation of additional sites by mTOR is required, most notably S65 and T70. The differentially phosphorylated isoforms of 4E-BP1 can readily be separated by SDS-PAGE and analyzed with phosphosite-specific antibodies. In asynchronously growing control cells 4E-BP1 appeared as a doublet of which the highest band was most intense (Figure 3a, lane 6). In Rheb-deficient cells 4E-BP1 antibodies recognized three distinct bands, of which the upper two were strongest (lane 1). Treatment with rapamycin completely shifted 4E-BP1 to the two fastest migrating species in Rheb-deficient cells, indicative for de-phosphorylation of the protein (lane 5). In control cells a fraction of 4E-BP1 remained in the upper band (lane 10), indicating that phosphorylation of 4E-BP1 is more resistant to rapamycin treatment compared to Rheb-deficient cells. PP242 treatment increased the mobility of 4E-BP1 in both cell lines (lanes 2-4 and 7-9). Probing the same lysates with phospho-specific T37/T46 anti-4E-BP1 antibody showed that PP242 eliminates phosphorylation at T37 and T46 in either cell line, while rapamycin was much less effective in blocking phosphorylation of these sites (compare lanes 2-4 with 5 and 7-9 with 10). This is in accordance with earlier publications [38] [39] [40]. Based on literature [41], we expected the upper band of 4E-BP1 in Rheb-deficient cells to represent a fraction of 4E-BP1 phosphorylated at S65 and/or T70. Phospho-specific antibodies for these sites indeed recognized bands at the expected size that disappeared following treatment with rapamycin or PP242. Phosphorylation of 4E-BP1 at T46 is strongly decreased in Rheb-deficient cells as demonstrated with a non-phospho-T46-specific antibody. Both rapamycin and PP242 induced a further increase in the levels of non-phospho-T46. These data again demonstrate that mTOR activity is decreased, but not absent, in Rheb-deficient cells compared to control cells. To rule out that the observed mTORC1 activity resulted from mutations during the immortalization process, we generated a second series of Rheb-deficient MEFs starting with MEFs that were immortalized by expression of SV40 large T. Also in these cells diminished mTORC1 activity was detectable as judged from phosphorylation of mTORC1 substrates like S6K and 4E-BP1 (Figure S4a and S4b). Furthermore, mTORC1 activity could also be induced by serum and TPA in a rapamycin sensitive manner in these cells (Figure S4a-c). 

Next, we investigated if physiological stimuli would modify 4E-BP1 phosphorylation in Rheb-deficient MEFs. Stimulation of serum starved Rheb-deficient cells with serum or insulin had little effect on the motility of 4E-BP1 (Figure 3b, compare lane 1 with 2-3 and lane 8 with 9-10). However, depletion of amino acids clearly decreased phosphorylation of 4E-BP1 even more than rapamycin treatment, as judged from the increase in the fastest migrating 4E-BP1 isoform (lane 4 versus 7 and 11 versus 14 in the 4E-BP1 panel). Add-back of amino acids did not restore 4E-BP1 phosphorylation (lane 4 versus 5), which is in line with the observations above that amino acids cannot rapidly activate mTORC1 in these cells (Figure 1c, lane 3 and 6). On the other hand, serum stimulation did restore phosphorylation of 4E-BP1 after amino acid deprivation (Figure 3b, lane 4 versus 6). In contrast, 4E-BP1 phosphorylation occurred in response to both amino acids and serum in wild type cells (lane 11 versus 12-13).

The phosphorylation status of 4E-BP1 is predictive for its association with eIF4E, whose activity is blocked by 4E-BP1 binding. Binding of 4E-BP1 to eIF4E was measured by isolating eIF4E with ^M7^GTP-sepharose beads. In accordance with the data above, we find that in asynchronously growing Rheb-negative cells a much larger fraction of 4E-BP1 is associated with eIF4E than in control cells (Figure 3c, lane 10-12). This difference is also seen when cells are serum starved (lane 4 versus 1 and 7). Rapamycin increases the fraction of eIF4E-bound 4E-BP1 in both Rheb positive (lane 5) and negative cell lines (lane 2 and 8), but the amount is still higher in the latter cells. Consistent with the more pronounced effect of amino acid starvation on T37/T46 phosphorylation of 4E-BP1, downregulation of mTORC1 activity by amino acid depletion results in more equal levels of eIF4E-bound 4E-BP1 in control and Rheb-deficient cells (compare lanes 3, 6 and 9). In summary, we conclude that the absence of Rheb diminishes 4E-BP1 phosphorylation and increases its association with eIF4E. As seen for other mTOR substrates, residual levels of phosphorylation are diminished by rapamycin or PP242 treatment. Increased mTORC1 activity is only seen after serum stimulation, but not following replenishment with amino acids or stimulation with insulin. 

**Figure 4:**
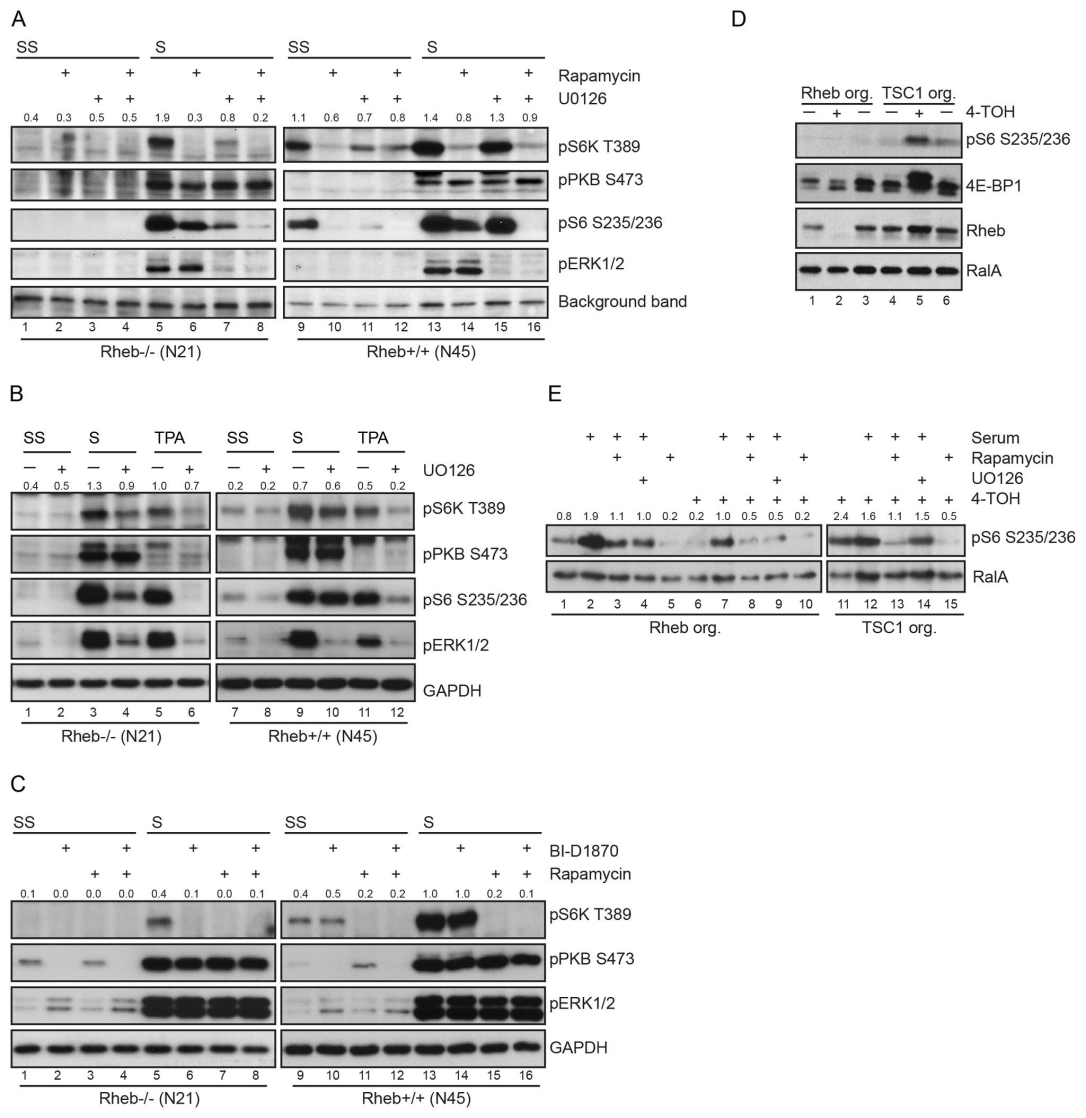
Effect of pharmacological inhibitors on mTORC1 activity in Rheb-deficient cells and wild-type cells. **a**. Rheb-deficient (N21) or control cells (N45) were serum starved overnight and treated with U0126 (10 µM), rapamycin (50 nM) or a combination of both inhibitors for one hour before harvesting. Inhibitors were added 1 hour before serum stimulation. Total lysates were analyzed by Western blotting with the antibodies indicated on the right. A background band was used as a loading control. **4b**. Effect of TPA on S6K phosphorylation in Rheb-deficient (N21) and control cells (N45). Cells were serum starved overnight and pretreated with U0126 for 1 hour or not. Cells were stimulated with serum or TPA for 90 or 30 minutes, respectively. Total cell lysates were analyzed by probing Western blots with the antibodies indicated on the right. **4c**. As 4A, but now with the RSK-inhibitor BI-D1870. In all cases immunoblots shown are representative of observations for at least two experiments. **4d**. Small intestinal organoids with floxed alleles of either Rheb or Tsc1 were grown for seven days after overnight incubation with vehicle or tamoxifen (4-TOH) as indicated and analyzed for the proteins indicated on the right. **4e**. Small intestinal organoids with floxed alleles of either Rheb (lane 1 to 10) or Tsc1 (lanes 11 to 15) were grown for seven days after overnight incubation with vehicle or tamoxifen (4-TOH) as indicated and left untreated (lanes 1, 5, 6, 10, 11, 15) or stimulated for 90 minutes with serum. Pretreatment with 50 nm rapamycin (lanes 3, 5, 8, 10, 13 and 15) or 10 µM U0126 (lanes 4, 9, 14). Numbers on top of immunoblots represent densitometric ratio of pS6 S235/236 over RalA. Numbers indicate the ratio of pS6K T389 relative to GAPDH in a, b and c and pS6 relative to RalA in e.

We next wished to delineate the signaling pathway via which serum can activate mTORC1 in Rheb-deficient cells by using a panel of pharmacological inhibitors. PI-3 kinase inhibitors like wortmannin and PI-103 efficiently inhibited mTORC1 (data not shown). Since we had noticed that serum, in contrast to insulin, significantly activates ERK in our fibroblasts, we blocked ERK activation with the MEK inhibitor U0126 (Figure 4a). U0126 clearly reduced T389 phosphorylation of S6K in Rheb-deficient cells (lane 5 versus 7). In wild type cells, U0126 had barely any effect (lane 13 versus 15).

Previously, a role for ERK and/or RSK in mTORC1 activation has been documented, either via inactivation of TSC2 [42][43] and/or via direct phosphorylation of Raptor [44] [15] [16]. First, we tested if specific activation of MEK/ERK with TPA could substitute for serum. Indeed, TPA activated mTORC1 (Figure 4b, lane 1 versus 5) suggesting that MEK/ERK activity is sufficient.

Since ERK can act upstream of RSK, the effect of the RSK inhibitor BI-D1870 on S6K phosphorylation by serum was tested. BI-D1870 had a profound effect on T389 phosphorylation of S6K in Rheb-negative cells (Figure 4c, lane 5 versus 6), indicating that it may be required. In contrast, S6K phosphorylation at T389 was largely insensitive to BI-D1870 in wild type cells (lane 13 versus 14). We noted however, that S473 phosphorylation of PKB was also affected in serum starved cells (compare lane 1 with 2 and 9 with 10) or asynchronously growing cells (data not shown), indicating that BI-D1870 may target other kinases than RSK. This has also been reported by others [45][46]. We wondered if genetic deletion of Rheb woud have the same effect in other cell types. To this end, we generated small intestinal organoids from mice with either a floxed allele of Rheb or TSC1 in combination with tamoxifen-inducible Cre recombinase [47]. In these organoids, deletion of Rheb decreased mTORC1 signaling, whereas TSC1 had the opposite effect (Figure 4d). Serum stimulation of Rheb-positive and Rheb-negative organoids revealed that loss of Rheb again lowered, but did not abolish S6 phosphorylation. Phosphorylation was sensitive to rapamycin, demonstrating the involvement of mTORC1 (Figure 4e). Also here U0126 had a profound effect.

**Figure 5:**
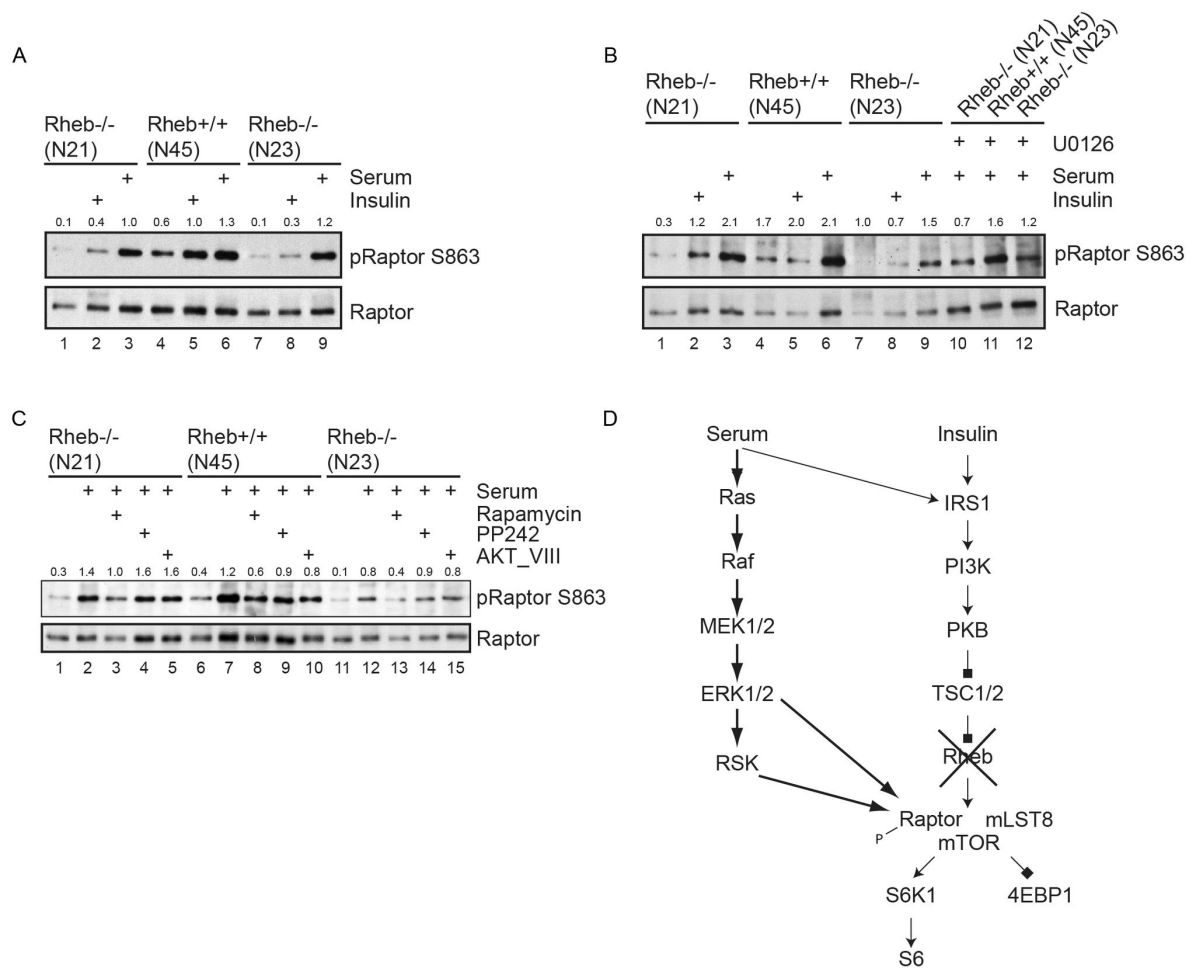
Analysis of Raptor phosphorylation. **5a**. Rheb-deficient (N21, N23) or control cells (N45) were serum starved overnight and stimulated for 30 minutes with insulin or 90 minutes with serum. Endogenous Raptor was immuno-precipitated and Western blots were probed for S863 phosphorylation (upper panel). Hereafter, blots were stripped and probed for total Raptor levels. A representative example from three experiments is shown. **5b** Rheb-deficient (N21, N23) or control cells (N45) were serum starved overnight and stimulated for 30 minutes with insulin or 90 minutes with serum or pretreated with 10 µM U0126 before serum stimulation. Endogenous Raptor was immuno-precipitated and Western blots were probed with a pS863 Raptor antibody (upper panel) and reprobed for Raptor (lower panel). A representative example from two experiments is shown. In all panels numbers above the blot indicate the ratio of Raptor pS863 relative to Raptor. **5c**. Rheb-deficient (N21, N23) or control cells (N45) were serum starved overnight and either left untreated or stimulated for 90 minutes with serum. Where indicated, cells were pretreated with rapamycin (50 nM), PP242 (2 µM) or the PKB-inhibitor AKT_VIII (10 µM) for 60 minutes. Hereafter, endogenous Raptor was immuno-precipitated and analyzed under 5a. The immunoblots shown are representative of observations for at least two experiments. A representative example from two experiments is shown. **5d**. Schematic representation of the mechanism of Rheb-independent mTORC1 activation. Arrows represent activation, squares indicate inhibition.

In the absence of Rheb, Raptor is a likely entrance point for stimulatory input on mTORC1. RSK has been reported to phosphorylate a cluster of conserved sites (S719, S721, S722) that are recognized by a phospho-PKB-substrate antibody [15]. In addition, ERK has been reported to phosphorylate S863, which functions as a priming phosphorylation required for further Raptor phosphorylation [16,44]. We therefore immunoprecipitated endogenous Raptor from Rheb-negative cells that had been serum starved. Insulin and serum stimulation caused a very mild increase in Raptor phosphorylation as detected with the phospho-PKB-substrate antibody suggesting that phosphorylation of these sites does not explain the differential effects on mTORC1 activity by these stimuli. A very similar pattern of phosphorylation was seen in Rheb-positive cells (Figure S5, lane 4 versus 5 and 6). S863 phosphorylation of Raptor showed a weak and variable response following insulin treatment in Rheb-deficient cells (Figure 5a, lane 2 and 8). In contrast, the effect of serum was strong and robust in both Rheb-deficient and control cells (lane 3, 6 and 9), consistent with the strong activation of ERK by serum. This induction was partially inhibited by U0126 (Figure 5b, lane 10-12). Since S863 has also been reported as a direct target site for mTORC1, we tested the effect of various inhibitors on serum stimulated S863 phosphorylation. The PKB inhibitor AKT_VIII only had an inhibitory effect in Rheb-proficient cells, as would be expected on the basis of the reported inhibitory action of PKB on TSC2 (Figure 5c, lane 2 versus 5, 7 versus 10 and 12 versus 15). Rapamycin lowered S863 phosphorylation in both Rheb-negative and -positive cells (lane 2 versus 3, 7 versus 8 and 12 versus 13). However, PP242 did not affect phosphorylation of this site in Rheb-deficient cells (lane 2 versus 4 and 12 versus 14) and only partially in Rheb-positive cells (lane 7 versus 9), suggesting that rapamycin may interfere in S863 phosphorylation by destabilizing mTORC1 rather than by inhibiting mTOR. Together, these data are consistent with the notion that phosphorylation of S863 via ERK may contribute to serum stimulated mTORC1 activity (Figure 5d). 

**Figure 6:**
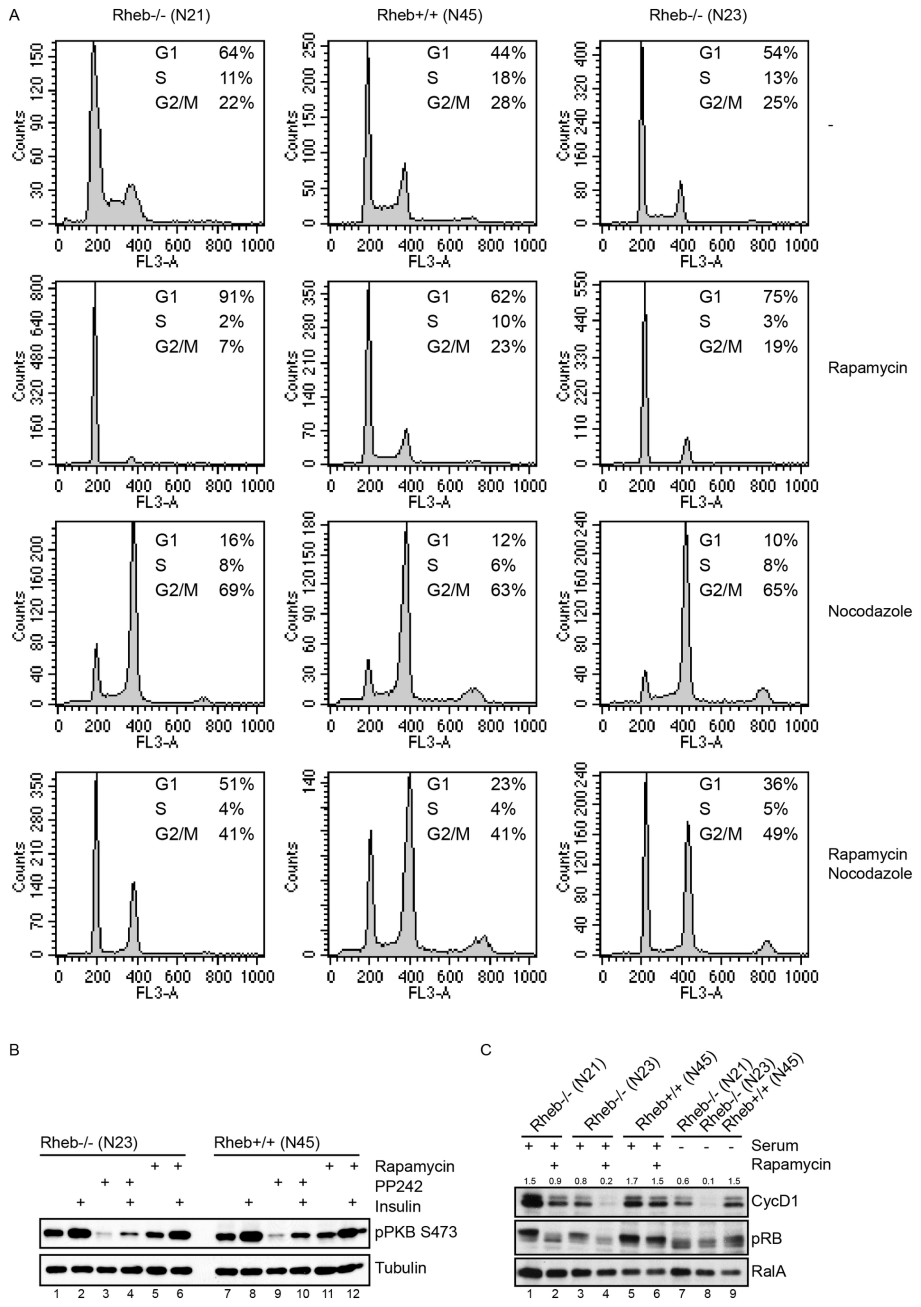
Effect of Rapamycin on cell cycle profile of Rheb-deficient (N21, N23) or control cells (N45). **6a**. Cells were grown in the absence or presence of 50 nM rapamycin, 250 ng/ml nocodazole or a combination thereof for 18 hours. Cell cycle profiles were determined as described under methods. The cell cycle profiles shown are representative of observations for two experiments. **6b**. Rheb-deficient (N23) or control cells (N45) were grown overnight in the presence of 1.0 µM PP242 or 50 nM rapamycin on mTORC2 activity. Subsequently, cells were stimulated with insulin for 30 minutes and total lysates were analyzed by Western blotting using the antibodies indicated on the right. The immunoblots shown are representative of observations for two experiments. 6c. Rheb-deficient (N21, N23) or control cells (N45) were grown overnight in the presence of 50 nM rapamycin or were serum starved for 24 hours. Total lysates were analyzed by Western blotting using the antibodies indicated on the right. The immunoblots shown are representative for two experiments. Numbers on top represent the densitometic ratio of cyclin D1 over RalA.

Finally, the physiological relevance of mTOR activity in Rheb-deficient cells was investigated by studying cell cycle profiles following overnight treatment with rapamycin of cells grown in the continuous presence of serum (Figure 6a). A clear increase in cells in G1 (44 to 62%) was seen in control cells at the cost of cells in S phase (18 to 10%) and to a lesser extent M phase (28 to 23%). The proportion of cells in G1 in untreated Rheb-deficient cells was higher than that of control cells (64 and 54 instead of 44%), but also here a rapamycin-induced increase was observed (64 to 91% and 54 to 75%). This change in cell cycle distribution was even more pronounced when we treated cells with nocodazole so that cycling cells would accumulate in the G2/M phase, unless they had been arrested in G1. Under these circumstances a two-fold increase in G1 was seen in control cells and a threefold increase in Rheb-deficient cells. Since long-term rapamycin treatment can affect mTORC2 function [48], we investigated phosphorylation of PKB by insulin. Both in untreated and rapamycin-treated cells, a clear induction of S473 of PKB was seen, independent of the Rheb status of cells (Figure 6b, compare lane 1 with 2 and 6 and lane 7 with 8 and 12). In contrast, overnight treatment with PP242 interfered in PKB phosphorylation (compare lane 2 with 4 and 8 with 10). This demonstrates that the observed cell cycle effect of rapamycin is mediated via inhibition of mTORC1 and not mTORC2. We used Western blotting to see if we could identify cell cycle regulators that were affected by overnight rapamycin treatment. Whereas we did not observe changes when cell lysates were probed with antibodies against p27 or p16 (data not shown), a pronounced decrease in cyclinD1 levels was noticed in Rheb-deficient cells. Consistent with this observation, phosphorylation of retinoblastoma decreased more in Rheb-deficient cells as compared to control cells (Figure 6c). Clearly, these data show that the remaining mTORC1 activity in Rheb negative cells is important for cell cycle progression.

## Discussion

The current study using Rheb-deficient MEFs confirms previous studies that Rheb is required for rapid and strong activation of mTORC1 upon stimulation of serum starved cells with insulin or amino acid replenishment of amino acid-deprived cells. Surprisingly, considerable mTORC1 activity is present in Rheb-deficient cells when grown in serum. A compensatory role for RhebL1 in mTORC1 activation is highly unlikely on the basis of our RNAi studies. Also, RhebL1 did not compensate for Rheb in genetic studies in mice [35]. 

How can we reconcile mTORC1 activity seen in Rheb-deficient cells when grown in serum with the lack of mTORC1 signaling following insulin stimulation? In contrast to insulin, serum activates ERK in our fibroblasts. Blocking ERK or its downstream target RSK with pharmacological inhibitors strongly affects the residual mTORC1 activity in Rheb-deficient cells. Since phosphorylation of Raptor at multiple residues by RSK [15] or ERK [16] has been shown to positively regulate mTORC1, we favor a model in which Raptor phosphorylation by these kinases is sufficient for a basal level of mTORC1 in the absence of Rheb. In the case of ERK, these sites include S863 that acts like a priming site required for further phosphorylation of Raptor. Indeed, we confirm that Raptor is phosphorylated at S863 by serum stimulation. Intriguingly, mTOR itself also phosphorylates S863 *in vitro* and *in vivo* and is required for the Rheb-mediated increase of S6K phosphorylation seen in overexpression studies [17,44]. This is consistent with our observation that the mTOR inhibitor PP242 affects S863 phosphorylation in Rheb–positive cells, but not in cells lacking Rheb. Thus, mTORC1 may have a dual input via Raptor S863 phosphorylation only one of which is Rheb-mediated. It should be stressed however, that multiple studies indicate that the complete phosphorylation pattern of Raptor is instrumental for mTORC1 activity rather than just S863 phosphorylation. Indeed, since mTORC1 activity is BI-D1870 sensitive, RSK-mediated Raptor phosphorylation is most likely also required for the basal level of mTORC1 activity in the absence of Rheb. We think that Rheb-independent mTORC1 activity is not restricted to MEFs given our observations in small intestinal organoids lacking Rheb and RNAi experiments in A549 cells. In addition, Fonseca et al. noticed very limited TSC2 phosphorylation during TPA-induced mTORC1 activation in HEK293 cells and suggested the existence of a TSC2-independent mechanism [46].

The molecular mechanism via which Rheb activates mTORC1 is still incompletely understood. From *in vitro* mTOR kinase assays it is clear that the association of Rheb with mTORC1 is not required for mTOR to be active. Rheb is not present in detectable amounts in immunoprecipitates used for mTOR kinase assays due to its low affinity for mTOR ([20], our unpublished observations). Rheb may activate mTOR kinase activity via direct binding [49] and this effect has been mimicked in *in vitro* mTORC1 kinase assays [20,50]. Alternatively, Rheb may enhance substrate binding to mTORC1, which is supported both by *in vivo* [51,52] and *in vitro* [53] evidence. Finally, Rheb may compete with mTORC1-inhibitory proteins like PRAS40 [19] [20] or possibly FKBP38 [54] [50] [53]. Unfortunately, we and others have not yet been able to find a specific effect of GTP-bound Rheb in *in vitro* mTORC1 assays (data not shown; see also 52). 

The fact that amino acid depletion has a profound effect on mTORC1 activity in Rheb-deficient cells seems at odds with the prevalent model of regulation of mTORC1 by amino acids. In this model, dimeric Rag GTPases are activated by amino acids and target mTORC1 from the cytoplasm to lysosomes where Rheb is localized [55] [25] [24]. Apparently, a parallel system operates to downregulate mTORC1 when cells are starved for amino acids. One option is that mTORC1 downregulation by amino acids involves activation of phosphatase PP2A by association with PR61ε, leading to dephosphorylation of MAP4K3 [56]. Alternatively, decreased activity of the class III PI-3 kinase VPS34 underlies the effect of amino acid depletion in Rheb-deficient cells. VPS34 produces PtdIns(3)P in an amino acid-dependent manner [23] [57]. Recently, PtdIns(3)P generated by VPS34 was shown to target PLD1 to lysosomes, where local production of phosphatidic acid could activate mTOR [58]. Interestingly, VPS34 negative MEFs resemble our Rheb deficient MEFs in that they do not acutely increase mTORC1 activity upon amino acid stimulation. Furthermore, when grown in the presence of serum VPS34 negative MEFs have normal levels of mTORC1 activity [59], demonstrating that the requirement for upstream activators of mTORC1 is dependent on the precise nature of the stimulus.

The TSC/Rheb/mTORC1 pathway is essential for rapidly adapting cellular and organismal metabolism to changes in nutrients and growth factors (e.g. [29] [60]). In addition, this pathway functions in myelination of the brain [35]. We demonstrate here a role for Rheb-independent mTORC1 activity in cell cycle progression under normal growth conditions. mTOR-mediated cell proliferation in MEFs is known to depend on repressing 4E-BPs [4]. The decreased mTOR activity in Rheb-deficient cells results in a significant increase in non-phosphorylated 4E-BP1 and a concomitant increase in the fraction of eIF4E-associated 4E-BP1. Remarkably, this has no major impact on cell proliferation since growth rates do not differ significantly between Rheb-negative and control cells. Rapamycin further increases non-phosphorylated 4E-BP1 and this correlates with an increase in cells in the G1-phase of the cell cycle. This results in a decreased expression level of cyclin D1 and diminished phosphorylation of retinoblastoma, especially in Rheb-deficient cells. A similar effect of rapamycin has previously been described [61] Since deletion of Rheb results in embryonic lethality in mid-gestation, which is much later than seen after disruption of another mTORC1-specific protein, Raptor [33], it is reasonable to assume that also during early embryogenesis mTORC1 is partially active in the absence of Rheb to drive cell cycle progression. This may seem implausible in light of all studies focusing on insulin-mediated mTORC1 activity, but is less surprising from an evolutionary point of view. Our previous evolutionary analysis of the mTOR pathway showed that Rheb was most likely present in the last eukaryotic common ancestor. However, during the emergence of e.g. green plants and algae Rheb has been lost, while mTORC1 proteins were conserved [26]. It will be interesting to see what the exact mode of activation is in species that contain mTORC1 proteins, but no Rheb. 

## Material and Methods

### Ethics statement

All experiments were approved by the local ethical committee for animal research (Dier Experimentele Commissie (DEC) Erasmus MC; Approval EMC 2467) and were in accordance with the institutional animal care and use committee guidelines.

### Antibodies and inhibitors

anti-phospho-T389-S6K, anti-phospho-S235/236-S6, anti-RAPTOR, anti-mTOR, anti-PRAS40, anti-phospho-S473-PKB, anti-S6K, anti-4EBP1, anti-phospho-T37/46-4EBP1, anti- non-phospho-T46-4EBP1, anti-phospho-ERK1/2, anti-pAMPK T172, anti-eIF4E and anti-IRS-1 were from Cell Signaling; anti-phospho-PRAS40 S183 from IBL-America; anti-phospho-T246-PRAS40 from Invitrogen BioSource; anti-Raptor was from the University of Dundee (J. Hastie), anti-PKB was home-made; anti-GAPDH and anti-phospho-Raptor S863 were from Santa Cruz; anti-Rheb was a monoclonal antibody generated by R.F. Lamb; anti-LAMP1 was a kind gift from Peter van der Sluijs. 

Rapamycin was purchased from ENZO Life Sciences, UO126 from Promega, BI-D1870 from Axon Medchem and PP242 and AKT_VIII from Sigma Aldrich. 

### Cell lines, tissue culture and transfections

To obtain MEFs, 13 day pregnant homozygously floxed Rheb mice in C57Bl/6 background [34] were decapitated under deep isoflurane anesthesia and the embryos were isolated to obtain MEFs. Spontaneously immortalized or large T immortalized MEFs with a floxed Rheb allele were maintained in Dulbecco’s modified Eagle’s medium (DMEM) supplemented with 10% FCS, 4 mM L-glutamine and pen/strep at 37 °C with 5% CO_2_. MEFs were infected with Ad-CMV-Cre adenovirus (Vector-Biolabs) in the presence of 4 μg/ml polybrene added to a 1:1 mixture of fresh medium and medium conditioned by wild type MEFs. Two days after infections cells were grown for two passages on fibronectin-coated dishes. Hereafter Rheb-negative, monoclonal cell lines were obtained by serial dilution in 96 well plates. A549 cells were grown in RPMI supplemented with 10% FCS, 4 mM L-glutamine and pen/strep at 37 °C with 5% CO_2_. Stimulation of cells was done after overnight serum starvation, eventually followed by amino acid depletion for two hours in custom-made amino acid free DMEM (Gibco Life Technologies). Serum stimulation was done with 10% fetal calf serum, final concentrations for insulin and TPA were 5 μg/ml and 100ng/ml, respectively. Stimulation with amino acids was done supplementing cells with the normal concentration of essential amino acids. Cells were pretreated with inhibitors for 60 minutes unless otherwise indicated. Concentrations used were: rapamycin (50 nM), UO126 and BI-D1870 (10 μM), PP242 as indicated. siRNA transfections were performed 72 and 48 hours before experiments with 50 nM ON-TARGETplus SMARTpools (Dharmacon Inc.) targeting indicated genes using Oligofectamine (Invitrogen). Small intestinal organoids were grown from Rheb^f/f^::Cag-CreERT^+^ and Tsc1^f/f^::Cag-CreERT^+^ [47] mice as described in [62]. Excision of respectively Rheb and TSC1 was induced by over-night treatment with tamoxifen. Seven days later, organoids were treated as indicated in the figure legends, washed in ice cold PBS and lysed in Laemmli sample buffer. 

### Cell lysates and immunoblotting

In all cases, cells were washed twice with ice cold PBS before scraping them in 1x Laemmli sample buffer. Western blotting was done using PVDF membrane. Blots were quantified with ImageJ.

### 7-methyl GTP-Sepharose pull-downs

7-Methyl GTP-Sepharose beads (GE Healthcare UK Limited; 15 μl/sample) were washed three times in M7-Lysis Buffer (100 mM KCl, 5 mM MgCl_2_, 0.5% TX-100, 20 mM Tris HCl (pH 7.5), 10% glycerol, 10 mM NaF, 1 mM Na_2_VO_4_ 0.1 µM Aprotinin and 1 µM Leupeptin). Cells were washed twice with cold PBS and lysed in M7-Lysis Buffer. After spinning at 4 °C, 14000 rpm for 10 minutes, Part of the lysate was mixed with a quarter volume 5x LSB. The rest was tumbled with beads for 45-60 minutes at 4 °C, washed 4 times with M7-Lysis Buffer and mixed with 40 μl of 1x LSB. 

### Quantitative real-time PCR

Expression of Rheb and RhebL1 mRNA was examined by reverse transcription of total RNA followed by real-time quantitative PCR on an ABI cycler using SYBR Green (ABI) and the oligonucleotides 5′-TACCGGTCTGTGGGAAAGTC-3′ (in exon 2), 5′-GCCCCGCTGTGTCTACAA-3′ (in exon 3) and 5′-TCCCCACCATATCCAACAAC-3′ (in exon 5) for Rheb and the oligonucleotides 5′-ACTCGTGTGCTATGCCACTG-3′ and 5′-GCGCAGAGAGTTAACCGAGT-3′ for RhebL1.

### FACS analysis

Cells were grown to 90% confluency and following trypsinization resuspended in medium collected from the corresponding plates. Following centrifugation (5’, 1200rpm), cells were washed once with PBS, resuspended in 300 μl PBS and 700μl 70% ethanol. After incubation at -20 °C for 45 minutes, cells were spun (5’, 1500 rpm) and resuspended in 500 μl PBS with 40 μg/ml Propidium Iodide and 10 μg/ml RNase. Following incubation at room temperature for 30 minutes in the dark, cell cycle analysis was performed on a BD FACSCalibur.

### Immunofluorescence

MEFs were grown to conﬂuency on glass coverslips and given fresh medium 16 hrs before fixation with 4% formaldehyde for 20 min. Cells were permeabilized with 0.25% Saponin for 10 minutes, blocked with 10% FCS in PBS with 0.25% Saponin for 30 minutes and incubated overnight with primary antibodies in PBS with 0.25% Saponin and 1% FCS. Incubation with secondary antibody in PBS with 0.25% Saponin and 1% FCS was done for 1 hour, and DAPI for 5 minutes. After washing cells were mounted and examined on a Zeiss Axioskop2 LSM510 confocal microscope.

### Statistics

Data are expressed as means ± st. dev. Significance was assessed by unpaired t-test in IBM SPSS Statistics 20 (Armonk, NY, USA). *P*-values less than 0.05 were considered as significant.

## Supporting Information

Figure S1 **Demonstration of complete absence of functional Rheb.** **S1a** Effect of short term infection of MEFs with a floxed allele of Rheb with Ad-CMV-Cre adenovirus. Total cell lysates were made of MEFs 96 hours after infection with increasing amounts of adenovirus in the presence of 2 or 4 mg polybrene/ml as indicated. Lysates were analyzed by Western blotting with the indicated antibodies. **S1b** Agarose gel with products of Q-PCR for Rheb mRNA from control cells (N45; lane 2 and 4) and Rheb-deficient (N23; lane 1 and 3) cells using primers from exon 2 and 3 (lane 1 and 2) or from exon 2 and 5 (lane 3 and 4). **S1c** Predicted truncated Rheb protein based on sequences of Q-PCR products from 1b, which matches prediction of targeting construct. Western blots of total cell lysates from Rheb-deficient cell lines (N21, N23) or Rheb-containing control cells (N45) probed with antibodies against proteins indicated.
(TIF)

Figure S2 **Localization of mTOR, Rheb and LAMP1 under various conditions in control and Rheb-deficient cells. S2a** Immunofluorescence of localization of mTOR (red), LAMP1 (green) or co-localization of both (merge, yellow) in control (N45) and Rheb-negative cells (N23) grown in the continuous presence of serum**. S2b** Quantification of the relative co-localization of mTOR and LAMP1 in control (N45) and Rheb-deficient (N23) cells as shown in Figure S2a. Immunofluorescence intensity was thresholded in Image-J and co-localization indices were determined with the following plugin; http://www.mbs.med.kyoto-u.ac.jp/imagej/index.html. **S2c**. Immunofluorescence of localization of Rheb (red), in control (N45, L12) and Rheb-negative cells (N23, L10) grown in the continuous presence of serum. **S2d**. Immunofluorescence of localization of mTOR (red), LAMP1 (green) or co-localization of both (merge, yellow) in control (N45) and Rheb-negative cells (N21) either starved for amino acids (-AA, top panel) or stimulated with amino acids (-AA+AA, bottom panel). **S2e**. Immunofluorescence of localization of mTOR (red), LAMP1 (green) or co-localization of both (merge, yellow) in control (N45) and Rheb-negative cells (N23) either serum starved (ss, top panel) or stimulated with insulin (+ins, bottom panel).
(TIF)

Figure S3 **Effect of energy stress and RhebL1 RNAi on the T389 phosphorylation in control and Rheb-deficient cells.** **S3a**. Cells kept in the presence of serum were treated with the agents indicated. Western blots with total lysates were probed with the antibodies indicated on the right. A representative example of two experiments is shown. Numbers on top of immunoblots indicate ratio Raptor S792 relative to Raptor. **S3b**. Western blot of total cell lysates from dishes that had been transfected with the indicated siRNA of Rheb-/- (N23) and Rheb+/+ (N45) cells. A representative example of two experiments is shown. Numbers on top of immunoblots indicate intensity of pS6K T389 relative to GAPDH. **S3c**. Quantification of the levels of RhebL1 RNA in Rheb-/- (N23) and Rheb+/+ (N45) cells as determined by Q-PCR. These were duplicates of the cells used in Figure S2b. **S3d**. Western blot of total cell lysates from dishes of A549 cells that had been transfected with the indicated siRNAs and either serum starved o/n, stimulated with insulin for 20 minutes, or grown in the continuous presence of serum (CS). Representative immmunoblots from two experiments are shown.
(TIF)

Figure S4 **Analysis of mTORC1 signalling under various conditions in Large T immortalized control and Rheb-deficient cells.** **S4a**. Large T immortalized MEFs that were either Rheb-deficient (L1, L10) or control cells (L12) were grown in the continuous presence of serum (CS), serum starved o/n (SS) and re-stimulated with either serum for 90 minutes (+S, 90’) or insulin for 20 minutes (+ins, 20’). **S4b**. Analysis of mTORC1 activity by Western blotting in total lysates of large T immortalized MEFs that were either Rheb-deficient (L1, L10) or control cells (L5). Cells were serum starved overnight and left untreated, stimulated with insulin for 30 minutes (Ins) or depleted for amino acids for two hours and then replenished with amino acids for 30 minutes (AA). **S4c**. Large T immortalized MEFs that were either Rheb-deficient (L10; upper panels) or control cells (L12; lower panels) were grown in the continuous presence of serum (CS), serum starved o/n (SS) and re-stimulated with either serum for 90 minutes (+S) or TPA for 90 minutes (TPA). Cells were treated with rapamycin (50 nM) for one hour before harvesting. Western blots of total cell lysates were probed with antibodies against proteins indicated. In all cases Western blots shown are representative for two experiments.
(TIF)

Figure S5 **Effect of insulin and serum stimulation on Raptor phosphorylation.** Rheb-deficient (N23) or control cells (N45) were serum starved overnight and stimulated for 30 minutes with insulin or 90 minutes with serum. Endogenous Raptor was immuno-precipitated and Western blots were probed with a phospo-PKB-substrate antibody (upper panel). Hereafter, blots were stripped and reprobed for total Raptor levels. A representative example of two experiments is shown. Numbers on top of immunoblots indicate ratio Raptor over pPKB substrate. Immunoblots are representative for two experiments.
(TIF)
