# Supplementary figures and images for: Mammalian Target of Rapamycin Complex I (mTORC1) Activity in Ras Homologue Enriched in Brain (Rheb)-Deficient Mouse Embryonic Fibroblasts
Source: PLoS One. 2013 Nov 26;8(11):e81649. doi: 10.1371/journal.pone.0081649 (PMC3841147; doi:10.1371/journal.pone.0081649)

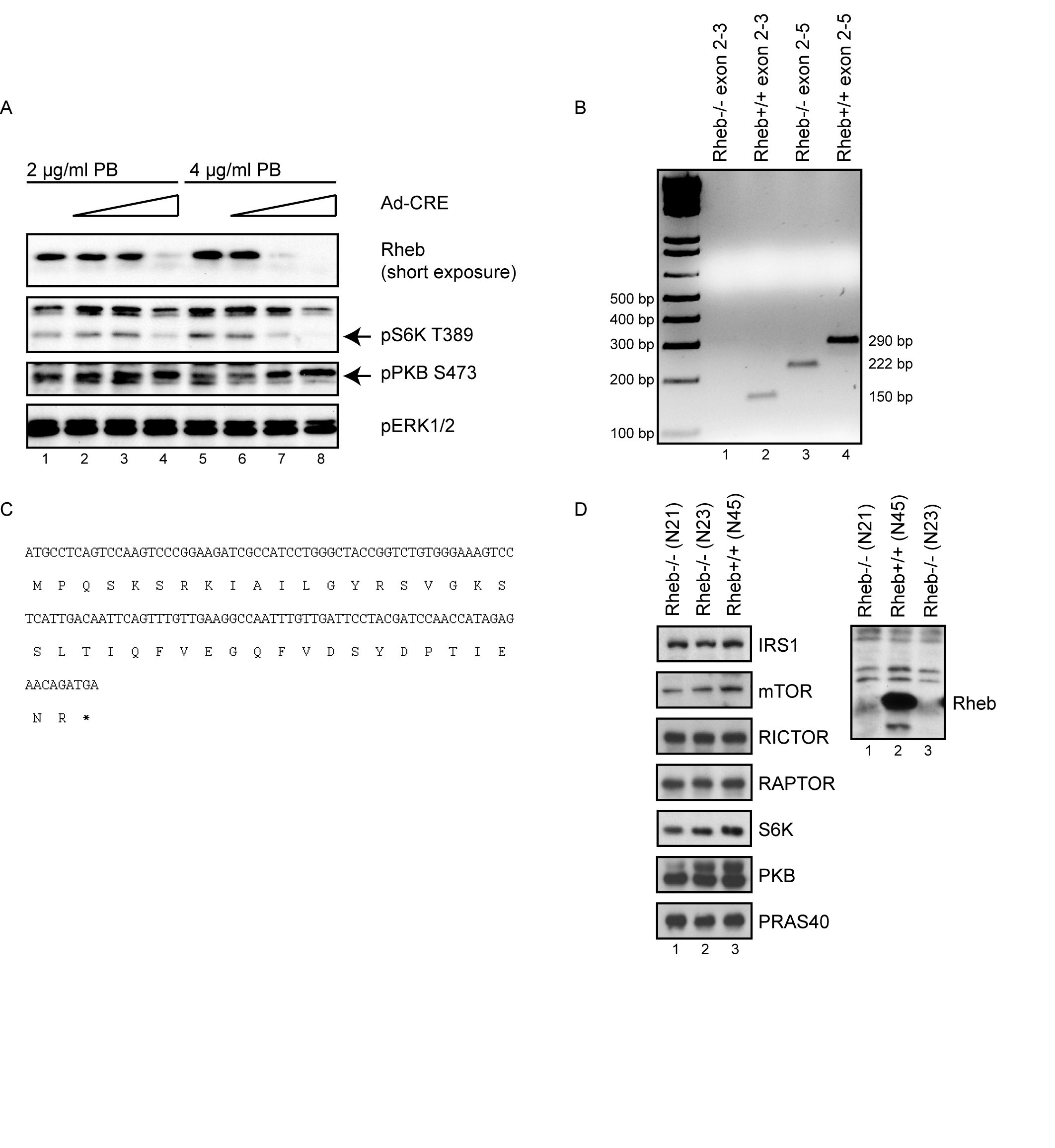

Supplement: Figure S1 — Demonstration of complete absence of functional Rheb. S1a Effect of short term infection of MEFs with a floxed allele of Rheb with Ad-CMV-Cre adenovirus. Total cell lysates were made of MEFs 96 hours after infection with increasing amounts of adenovirus in the presence of 2 or 4 mg polybrene/ml as indicated. Lysates were analyzed by Western blotting with the indicated antibodies. S1b Agarose gel with products of Q-PCR for Rheb mRNA from control cells (N45; lane 2 and 4) and Rheb-deficient (N23; lane 1 and 3) cells using primers from exon 2 and 3 (lane 1 and 2) or from exon 2 and 5 (lane 3 and 4). S1c Predicted truncated Rheb protein based on sequences of Q-PCR products from 1b, which matches prediction of targeting construct. Western blots of total cell lysates from Rheb-deficient cell lines (N21, N23) or Rheb-containing control cells (N45) probed with antibodies against proteins indicated. (TIF) [file pone.0081649.s001.tif]

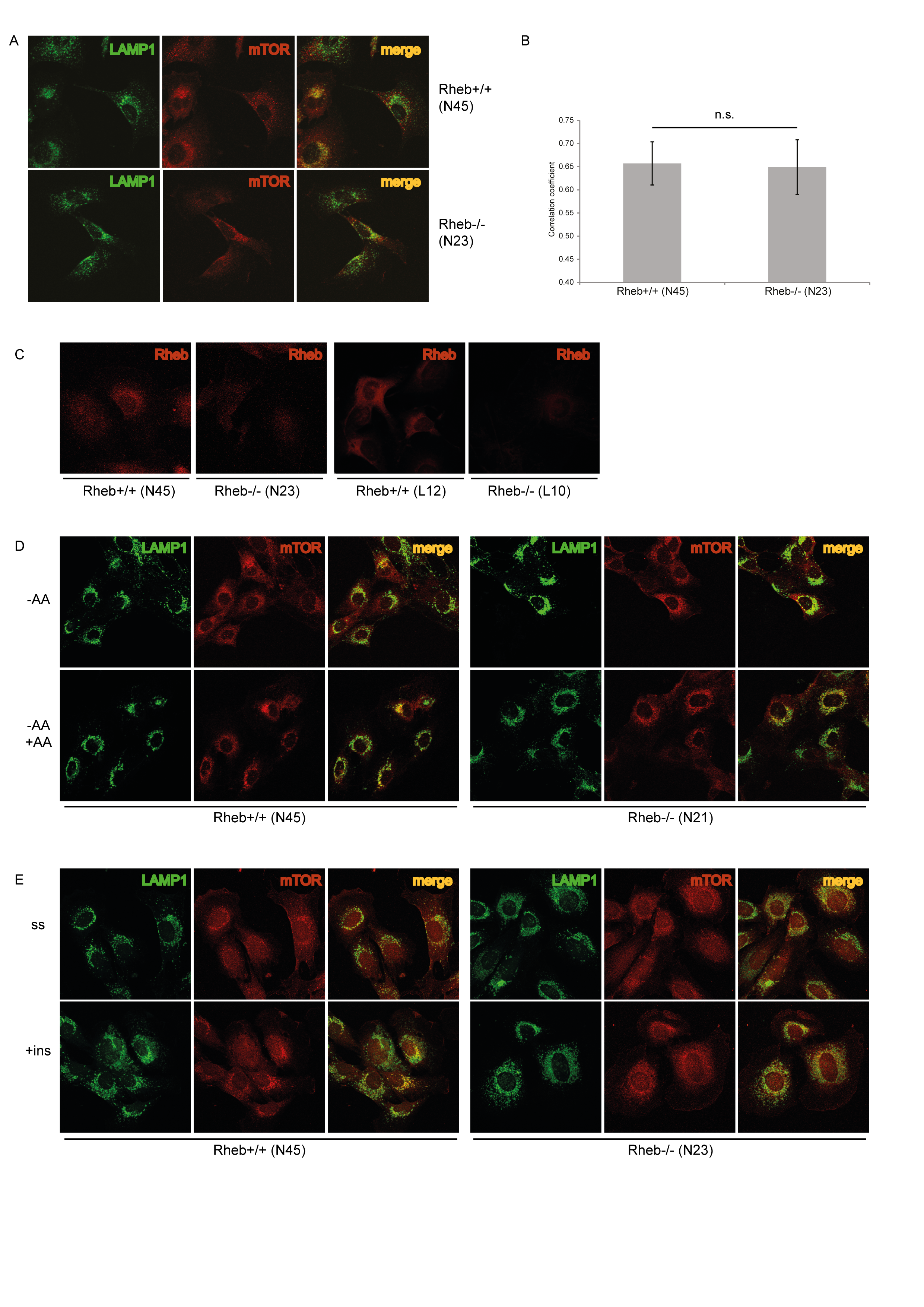

Supplement: Figure S2 — Localization of mTOR, Rheb and LAMP1 under various conditions in control and Rheb-deficient cells. S2a Immunofluorescence of localization of mTOR (red), LAMP1 (green) or co-localization of both (merge, yellow) in control (N45) and Rheb-negative cells (N23) grown in the continuous presence of serum. S2b Quantification of the relative co-localization of mTOR and LAMP1 in control (N45) and Rheb-deficient (N23) cells as shown in Figure S2a. Immunofluorescence intensity was thresholded in Image-J and co-localization indices were determined with the following plugin; http://www.mbs.med.kyoto-u.ac.jp/imagej/index.html. S2c. Immunofluorescence of localization of Rheb (red), in control (N45, L12) and Rheb-negative cells (N23, L10) grown in the continuous presence of serum. S2d. Immunofluorescence of localization of mTOR (red), LAMP1 (green) or co-localization of both (merge, yellow) in control (N45) and Rheb-negative cells (N21) either starved for amino acids (-AA, top panel) or stimulated with amino acids (-AA+AA, bottom panel). S2e. Immunofluorescence of localization of mTOR (red), LAMP1 (green) or co-localization of both (merge, yellow) in control (N45) and Rheb-negative cells (N23) either serum starved (ss, top panel) or stimulated with insulin (+ins, bottom panel). (TIF) [file pone.0081649.s002.tif]

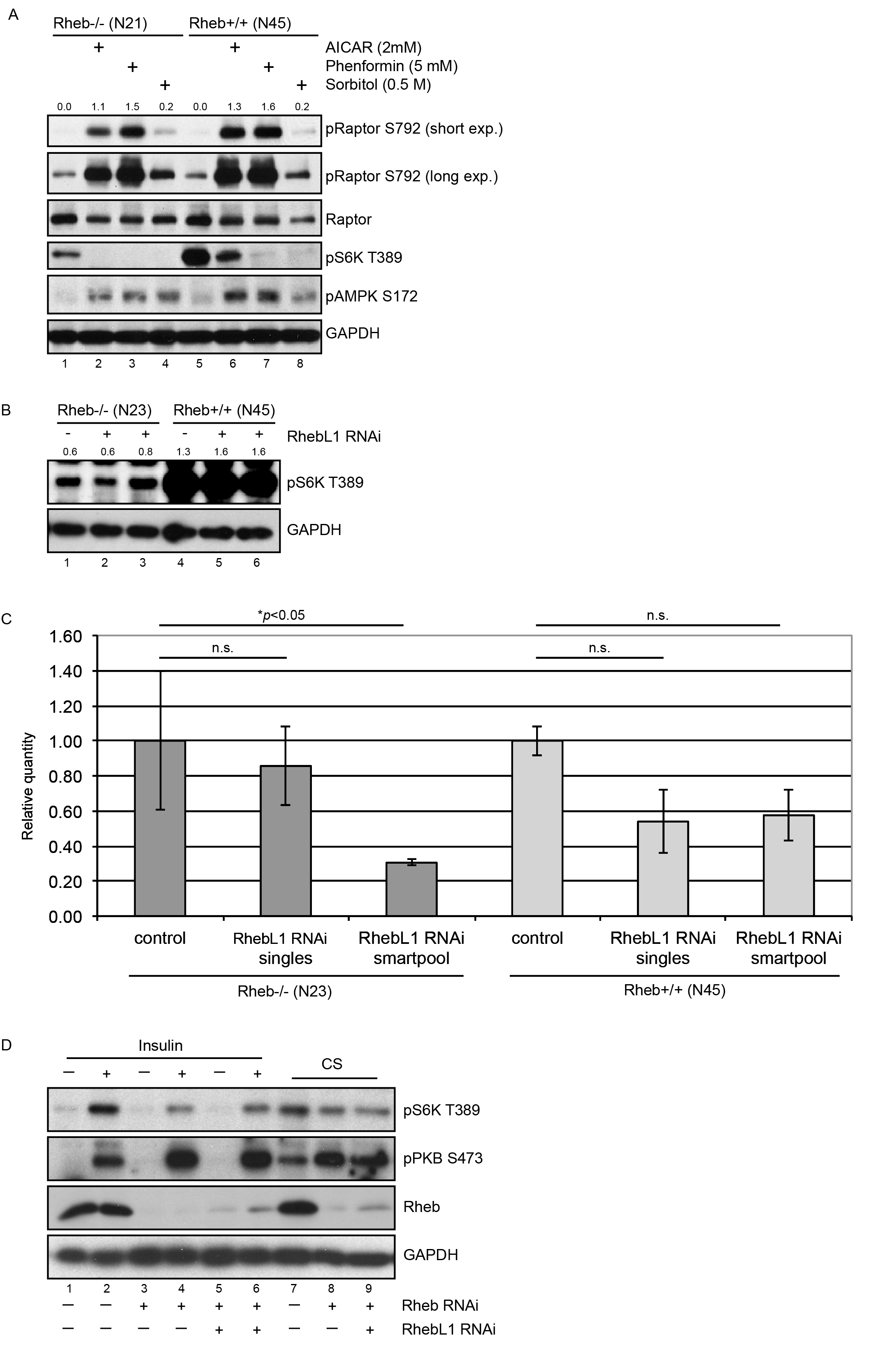

Supplement: Figure S3 — Effect of energy stress and RhebL1 RNAi on the T389 phosphorylation in control and Rheb-deficient cells. S3a. Cells kept in the presence of serum were treated with the agents indicated. Western blots with total lysates were probed with the antibodies indicated on the right. A representative example of two experiments is shown. Numbers on top of immunoblots indicate ratio Raptor S792 relative to Raptor. S3b. Western blot of total cell lysates from dishes that had been transfected with the indicated siRNA of Rheb-/- (N23) and Rheb+/+ (N45) cells. A representative example of two experiments is shown. Numbers on top of immunoblots indicate intensity of pS6K T389 relative to GAPDH. S3c. Quantification of the levels of RhebL1 RNA in Rheb-/- (N23) and Rheb+/+ (N45) cells as determined by Q-PCR. These were duplicates of the cells used in Figure S2b. S3d. Western blot of total cell lysates from dishes of A549 cells that had been transfected with the indicated siRNAs and either serum starved o/n, stimulated with insulin for 20 minutes, or grown in the continuous presence of serum (CS). Representative immmunoblots from two experiments are shown. (TIF) [file pone.0081649.s003.tif]

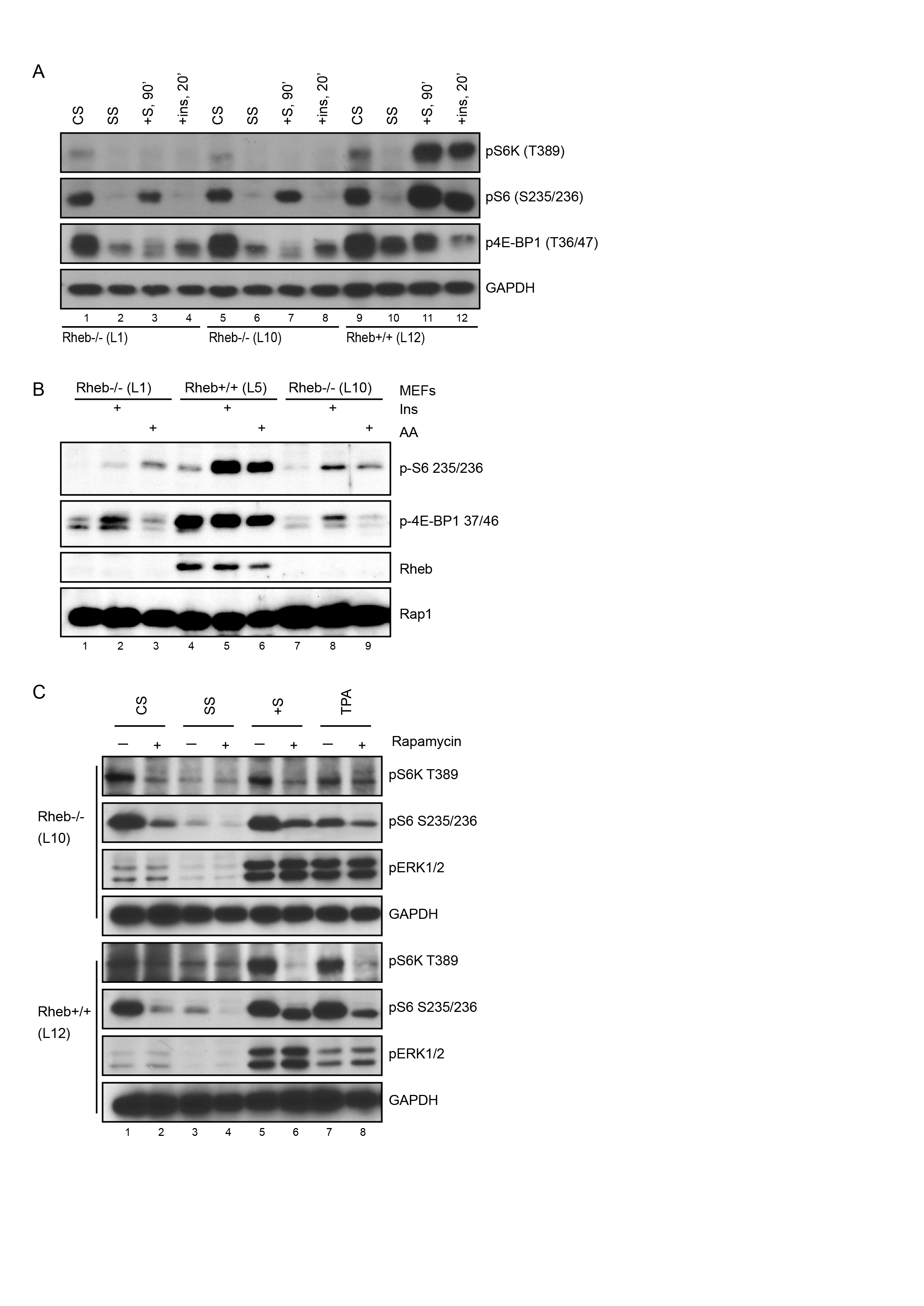

Supplement: Figure S4 — Analysis of mTORC1 signalling under various conditions in Large T immortalized control and Rheb-deficient cells. S4a. Large T immortalized MEFs that were either Rheb-deficient (L1, L10) or control cells (L12) were grown in the continuous presence of serum (CS), serum starved o/n (SS) and re-stimulated with either serum for 90 minutes (+S, 90’) or insulin for 20 minutes (+ins, 20’). S4b. Analysis of mTORC1 activity by Western blotting in total lysates of large T immortalized MEFs that were either Rheb-deficient (L1, L10) or control cells (L5). Cells were serum starved overnight and left untreated, stimulated with insulin for 30 minutes (Ins) or depleted for amino acids for two hours and then replenished with amino acids for 30 minutes (AA). S4c. Large T immortalized MEFs that were either Rheb-deficient (L10; upper panels) or control cells (L12; lower panels) were grown in the continuous presence of serum (CS), serum starved o/n (SS) and re-stimulated with either serum for 90 minutes (+S) or TPA for 90 minutes (TPA). Cells were treated with rapamycin (50 nM) for one hour before harvesting. Western blots of total cell lysates were probed with antibodies against proteins indicated. In all cases Western blots shown are representative for two experiments. (TIF) [file pone.0081649.s004.tif]

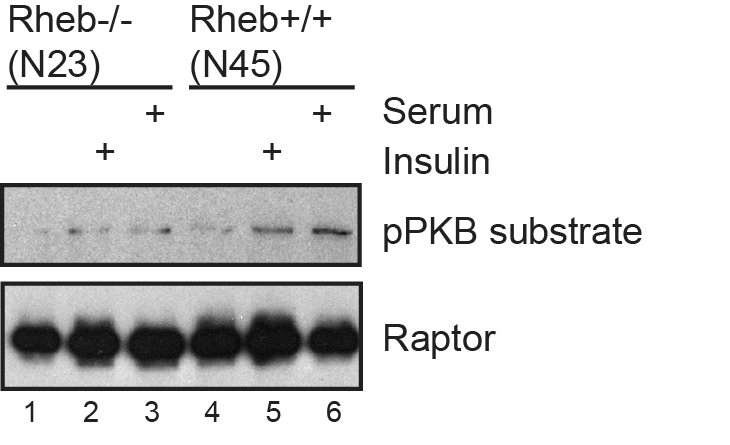

Supplement: Figure S5 — Effect of insulin and serum stimulation on Raptor phosphorylation. Rheb-deficient (N23) or control cells (N45) were serum starved overnight and stimulated for 30 minutes with insulin or 90 minutes with serum. Endogenous Raptor was immuno-precipitated and Western blots were probed with a phospo-PKB-substrate antibody (upper panel). Hereafter, blots were stripped and reprobed for total Raptor levels. A representative example of two experiments is shown. Numbers on top of immunoblots indicate ratio Raptor over pPKB substrate. Immunoblots are representative for two experiments. (TIF) [file pone.0081649.s005.tif]
